# Supplementary material for: Characterisation of a soil MINPP phytase with remarkable long-term stability and activity from Acinetobacter sp
Source: PLoS One. 2022 Aug 31;17(8):e0272015. doi: 10.1371/journal.pone.0272015 (PMC9432691; doi:10.1371/journal.pone.0272015)
Supplement: S1 File — (PDF) [file pone.0272015.s002.pdf]

Supporting information

Isolation and Characterisation of a Soil MINPP Phytase from Acinetobacter sp. with Remarkable Long-Term Stability and Activity.

[1]Gregory D. Rix, [1]Colleen Sprigg, [1]Hayley Whitfield, [1,2]Andrew M. Hemmings, [1]Jonathan D. Todd, and [1]Charles A. Brearley*

[1]School of Biological Sciences, University of East Anglia, Norwich Research Park, Norfolk NR4 7TJ

[2]School of Chemistry, University of East Anglia, Norwich Research Park, Norfolk NR4 7TJ

Methods including Table S1 (cloning primers used) and Table S2 (qPCR primers)

AC1-2 MINPP Nucleotide sequence

AC1-2 MINPP Amino acid sequence

Figure Legends

References

Figure S1. A Phylogram of Phytase Classes

Figure S2. Assignment of identity of $InsP_4$ products of AC1-2 MINPP action on $InsP_6$

Figure S3. Purification of $InsP_5$ fractions

Figure S4. Long-term stability of an impure, signal peptide-containing AC1-2 MINPP

Figure S5. Product profile of an impure, signal peptide-containing AC1-2

Methods

Genomic DNA extraction - Genomic DNA was isolated from a cell suspension grown from a single colony in LB. The cells were lysed with lysozyme. DNA was extracted with phenol-choroform, washed with 80% ethanol, air-dried and recovered in dH_2O.

Gateway Cloning - Primer sets 1/3 were used to clone the gene without the signal peptide, with added attB sites and 3C protease cleavage site, from genomic DNA. The attB-PCR fragment was cloned into pDONR207™ with BP Clonase™ (Invitrogen) to generate the entry clone, BP reaction. This reaction used 2 µL TE buffer, 1 µL BP clonase, 1 µL Template DNA and 1 µL pDONR 207 and was incubated at 25 °C for 3hr 30mins. After which, the BP reaction mixture was incubated with 0.5 µL Proteinase K for 10 mins at 37 °C and then transformed into E. coli DH5α cells with the appropriate antibiotics.

Table S1: **Primer sets used**. The 3C protease cleavage site is underlined, and those highlighted in grey are the attB1 and attB2 recombination sites.

Primer Set 1:	FOR:5'-CTGGAAGTTCTGTTTCAGGGCCCG…ATGAATATTTTATTTAAAACGACGAT-3'<br>REV: 5'-CAAGAAAGCTGGGTTTAA…TTGTATTTGATAGCACTGTTTC-3'
Primer Set 2:	FOR: 5'-GGGGACAAGTTTGTACAAAAAAGCAGGCTTCCTGGAAGTTCTGTT-3'<br>REV: 5'-GGGGACCACTTTGTACAAGAAAGCTGGGTT-3'
Primer Set 3:	FOR: 5'-CTGGAAGTTCTGTTTCAGGGCCCG…AACAATAATGACGATCAAGATG-3'<br>REV: 5'-CAAGAAAGCTGGGTTTAA…TTGTATTTGATAGCACTGTTTC-3'

The cloned gene was transferred into the entry vector, pDONR207 using BP clonase to create the Gateway entry clone. This entry clone was transferred into the destination vector pDEST17, which contains a 6X His-tag, using LR clonase and transformed into the expression host Rosetta 2 pLysS.

PCR protocol: initial denaturation, 95 °C, 3:00 (min:sec); denaturation, 95 °C, 0:20; annealing, 50 °C, 0:30; elongation 72 °C, 1:00; with 30 cycles and final elongation at 72 °C for 10:00.

Initial cloning with Primer Sets 1 and 2 (Table S1) yielded, on subsequent expression, mostly insoluble protein with low activity. Therefore, Primer Set 3 was designed to remove the first 19 amino acids corresponding to the signal peptide, SignalP 5.0 (Almagro Armenteros et al., 2019). Once purified, the resulting SDS PAGE band was sent for sequencing using Protein Mass Fingerprinting at the John Innes Centre (Norwich, UK) confirming the expression of the AC1-2 protein.

Successful transformations were confirmed using colony PCR and sequenced using internal attL primers, FOR 5'GCAGTTCCCTACTCTCGC 3' and REV 5' CATCAGAGATTTTGAGACAC 3' and a gene internal primer 5'-TGA TTT AGA AGC AAT GAT G-3. The DH5α cells were grown overnight, and plasmid DNA obtained using the QIAprep Spin Miniprep Kit. The LR reaction was then used to create the expression clone, pDON7207 using LR Clonase™ (Invitrogen). This reaction used 2 µL TE buffer, 1 µL LR clonase, 1 µL pDONR 207 and 1 µL pDEST17 and was incubated at 25 °C for 3hr30mins. The LR reaction mix was then transformed into E. coli DH5α cells. Successful transformation was confirmed using colony PCR and the plasmid DNA was transformed again into the expression host Rosetta 2 pLysS (Novagen).

Transformation - DH5α cells were transformed by standard heat shock protocol.

Protein purification - Single colonies from the Rosetta 2 pLysS AC1-2 clone were inoculated into 100 mL LB, shaken overnight at 180 RPM and 37 °C, then used to inoculate 4X500 mL LB. At OD600 > 0.5, IPTG was added to 0.1 mM and the cultures incubated at 16 °C overnight. Cells were centrifuged at 4500 RPM at 20 °C using a Beckman JLA 8.1 rotor in a J-20 centrifuge, resuspended in 25 mL binding buffer

containing cOmplete™ Protease Inhibitor Cocktail (LaRoche), Dnase, lysozyme and 50 µL EDTA (0.5 M pH 8). Cells were lysed by French Press, repeated 3 times, at 1000 psi, centrifuged for 45 minutes at 19,000 RPM at 4 °C in a Beckman JA-25.50 rotor in a J-20 centrifuge. The supernatant was passed through a 1 mL Histrap HP column on an ÄKTA pure protein purification. UV- absorbing fractions were pooled and concentrated to 1.5 mL using an Amicon® Ultra-15 centrifugal filter unit (30 kDA) cutoff). The concentrated protein was further purified on a HiLoad 16/600 Superdex 75 PG column. Fractions, selected by SDS PAGE, were pooled and concentrated to 1 mL (30 kDA cutoff). Protein was stored in 4 µM aliquots in different cryoprotectants: final concentration 25% (w/v) trehalose, 25% (w/v) trehalose and 1 mg/mL BSA, 25% (w/v) sucrose, 25% (w/v) sucrose and 1 mg/mL BSA, 25% (w/v) glycerol, 25% (w/v) glycerol and 1 mg/mL BSA, 1 mg/mL BSA, or gel filtration buffer in a 1:1 mixture. The protein was stored at -20 °C and thawed when necessary.

Protein Purification Buffers - Binding Buffer, 50 mM Tris-HCl pH 7.5, 300 mM NaCl, 20 mM Imidazole. Elution Buffer, 50 mM Tris-HCl pH 7.5, 300 mM NaCl, 500 mM Imidazole Gel Filtration Buffer, 50 mM Tris-HCl pH 7.5, 300 mM NaCl.

qPCR Primer Design - Primer efficiencies were calculated using a two-step qPCR protocol described below from a dilution series of cDNA, 1:1, 1:5, 1:10, 1:25, 1:100. The primer efficiency for primer sets 1 and 2 was calculated to be 105.4 and 97.5%, respectively. Primer set 1 was employed for LB and LB + $InsP_6$ comparison and primer set 2 for MM and MM + InsP6 comparison. The fold change of AC1-was calculated by the 2-ΔΔCt method, normalised to the housekeeping gene RecA. The concentration of $InsP_6$ and inorganic phosphate used were 1 mM and 365 µM, respectively.

qPCR Protocol - Initial denaturation 95 °C, 3:00 min; denaturation 95 °C, 0:03 min; annealing 60 °C, 0:30 min, 40 cycles. After the qPCR cycles were complete, a melt curve was generated to ensure only a single species had been amplified. 95 °C 0:15, Annealing 60 °C 1:00, Step and Hold (+0.3 °C increments) to 95 °C 0:15.

Table S2: Primer sets used for qPCR

	Position	Sequence (5' -> 3')	Primer Length (bp)
Forward Primer-1	82	CAACCAACGACATCACCTACAAC	23
Reverse Primer-1	221	CCATGACGCGCCACTAACT	19
Length	140 bp		
Forward Primer-2	1079	TATGCCTATTGAAGCCGCAAA	21
Reverse Primer-2	1278	CAGCATGGGCAAAACGTAAA	20
Length	200 bp		

AC1-2 MINPP DNA Sequence
>AC1-2.MINPP.1572.bases
atgaatattttatttaaaacgacgatgcttgcaacatcattatttcttgtggcctgcaac
aataatgacgatcaagatgatcaaccaacgacatcacctacaactcaatcaaagtattat
caaaccaaaacgccttaccaaccacaacaagatttaaaaagctatgaacaggcaccaaat
ggattccagccagtttttacagagttagtggcgcgtcatggttcaagaggtttatcaagt
ctcaaatatgatttagcactttataatttatggaagcaggcaaaagcagaaaatgcctta
acgccgttaggtgagcaattaggtgctgatttagaagcaatgatgaaagccaatattctg
ttaggttatggggtcgaaggcattcgccagtatggttatgggaatgaaaccatgacgggt
attttagagcaccgtggtattgcagatcggttattacaacgcttacccaatcttttaaat
ccgcaagcgggtatattggttcaaagttcaggcgtcgatcgtgcggtagatagtgctaaa
tttttcactgctgaactcattaaacaacaaccgcagctcaaagataaaattgttccagtc
tcttataccaatttatcgagtgaaagtgttccaagtgttatagatggcggagttgaccgt
tttaaactttattttcatagtttgaatgcggatgaagacttaacacagccactcagtgct
agtcagcagaaaatttatgatgcaagtcaggcctatcaagattttgaagaaaataataat
gacttagcacaaaagctggatgagttatcaaaaaatactcaagctgaaaaaactgctcag
actgtactcaacccgatttttaaagcagactttattaaaaaattgggtacagcgggttat
agctttagtaatacagggtcttttaccgtaacttcgcctaaaggtgagcaaattacggaa
aaaggtaaggggaaaaacactatcgccagtgcagttgatgcagcagcttatgtgtatgag
ctctattcgatttctggtggcatgaaagatgagctaaaaggtatcgattttgataaatat
atgcctattgaagccgcaaaattctatgctgaatttaatgatgccaatgatttctatgaa
aaaggcccaagtttcacagagtcaaatctggtcacgagtgaaattgcacaaggcttaaaa
caagatatgttccaacaagttgatgctgttgtgaataaagcccaaccttacaaagcggtt
ttacgttttgcccatgctgaaattattattcctctggccaccagcctagacttacataat
atgatgcaacctttacctctacgccagacttataactattcaacaagcacatggcgtgga
gaagtagtttcacctatggctgccaatgtacagtgggatatttatcaaaatggccaaggc
aatactttggtaaaaatgctttataacgaaaaagaaactttgtttaagtctgcatgtaac
tatgcgcgctatagcccaactagtttctactacgactacataaaattgaaacagtgctat
caaatacaatag

AC1-2 MINPP Protein Sequence
>AC1_2.MINPP.523.residues
MNILFKTTMLATSLFLVACNNNDDQDDQPTTSPTTQSKYYQTKTPYQPQQDLKSYEQAPN
GFQPVFTELVARHGSRGLSSLKYDLALYNLWKQAKAENALTPLGEQLGADLEAMMKANIL
LGYGVEGIRQYGYGNETMTGILEHRGIADRLLQRLPNLLNPQAGILVQSSGVDRAVDSAK
FFTAELIKQQPQLKDKIVPVSYTNLSSESVPSVIDGGVDRFKLYFHSLNADEDLTQPLSA
SQQKIYDASQAYQDFEENNNDLAQKLDELSKNTQAEKTAQTVLNPIFKADFIKKLGTAGY
SFSNTGSFTVTSPKGEQITEKGKGKNTIASAVDAAAYVYELYSISGGMKDELKGIDFDKY
MPIEAAKFYAEFNDANDFYEKGPSFTESNLVTSEIAQGLKQDMFQQVDAVVNKAQPYKAV
LRFAHAEIIIPLATSLDLHNMMQPLPLRQTYNYSTSTWRGEVVSPMAANVQWDIYQNGQG
NTLVKMLYNEKETLFKSACNYARYSPTSFYYDYIKLKQCYQIQ*

Supplementary Figure 1. A Phylogram of Phytase Classes. Seventeen Beta-propeller Phytases (BBPhy), ten Protein Tyrosine Phosphatase-like phytases (PTPLP), twenty-two Purple Acid Phytases, twenty one Histidine Acid Phytases (HAPhy) and twenty-seven Multiple Inositol Polyphosphate Phosphatase (MINPP) are compared.

Supplementary Figure 2. Assignment of identity of $InsP_4$ products of AC1-2 MINPP action on $InsP_6$.: HPLC separation of: A, Ins(1,3,4,6)P_4, In(1,3,4,5)P_4 and Ins(1,4,5,6)P_4 standards; **B**, products of incubation of AC1-2 MINPP with phytate ($InsP_6$); **C**, as B with added Ins(1,3,4,5)P_4 and Ins(1,4,5,6)P_4; **D**, an acid-hydrolysate of $InsP_6$. Note: Ins(1,3,4,6)P_4 and D/L-Ins(1,2,3,4)P_4 coelute on the similar CarboPac PA100 column (Chen and Li, 2003) and were not distinguished here on CarboPac PA200. The units and scales of panels (A-C) are identical. The HPLC gradient employed was modified from that of Whitfield *et al.* (2020), reaching 0.6 M methanesulfonic acid at 38 min.

Supplementary Figure 3. Purification of $InsP_5$ fractions. HPLC of: **A**, a desalted D-and/or L-Ins(1,2,3,4,5)P_5 [$InsP_5$ 4/6-OH] fraction generated by AC1-2 MINPP; **B**, a desalted D-and/or L-Ins(1,2,4,5,6)P_5 [$InsP_5$ 1/3-OH]//Ins(1,3,4,5,6)P_5 ($InsP_5$ [2-OH]) fraction; **C**, an acid-hydrolysate of phytate ($InsP_6$) showing resolution of all resolvable $InsP_5$s. The units and scales of panels (A-C) are identical.

Supplementary Figure 4. Long-term stability of an impure, signal peptide-containing AC1-2 MINPP. A, SDS-PAGE on a 12% gel of signal peptide-containing AC-2 MINPP after two-step, Ni-affinity and Sepharose, purification. Lanes labelled 1, 2, 3 and 4 show replicate aliquots of protein, flanked by molecular mass markers identified by mass (kDa) and separated by an empty lane. Protein in Lanes 3 and 4 was supplemented with 1 mM DTT. **B**, Enzyme activity of this 'impure' protein preparation over a period of 750 days. The protein was stored at a concentration of 4 µM in 25 mM Tris-HCl pH 7.5 150 mM NaCl, 25% w/v trehalose at 4 °C or ambient conditions (occasionally reaching 30-35 °C). Error bars show standard deviation of triplicate measurements.

Supplementary Figure 5. HPLC of products of phytase assays with an impure, signal peptide-containing AC1-2 MINPP. Degradation of phytate was followed by HPLC. The units and scales of panels (A-C) are identical.

References

Almagro Armenteros JJ, Tsirigos KD, Sønderby CK, Petersen TN, Winther O, Brunak S, et al. SignalP 5.0 improves signal peptide predictions using deep neural networks. Nat Biotechnol. 2019;37(4):420-423.

Chen Q-C, Li BW. Separation of phytic acid and other related inositol phosphates by high-performance ion chromatography and its applications. Journal of Chromatography A 2003;1018(7):41-52.

Whitfield H, White G, Sprigg C, Riley AM, Potter BVL, Hemmings AM, et al. An ATP-responsive metabolic cassette comprised of inositol tris/tetrakisphosphate kinase 1 (ITPK1) and inositol pentakisphosphate 2-kinase (IPK1) buffers diphosphoinositol phosphate levels. Biochem J. 2020;477(14):2621-2638.

Figure S1.

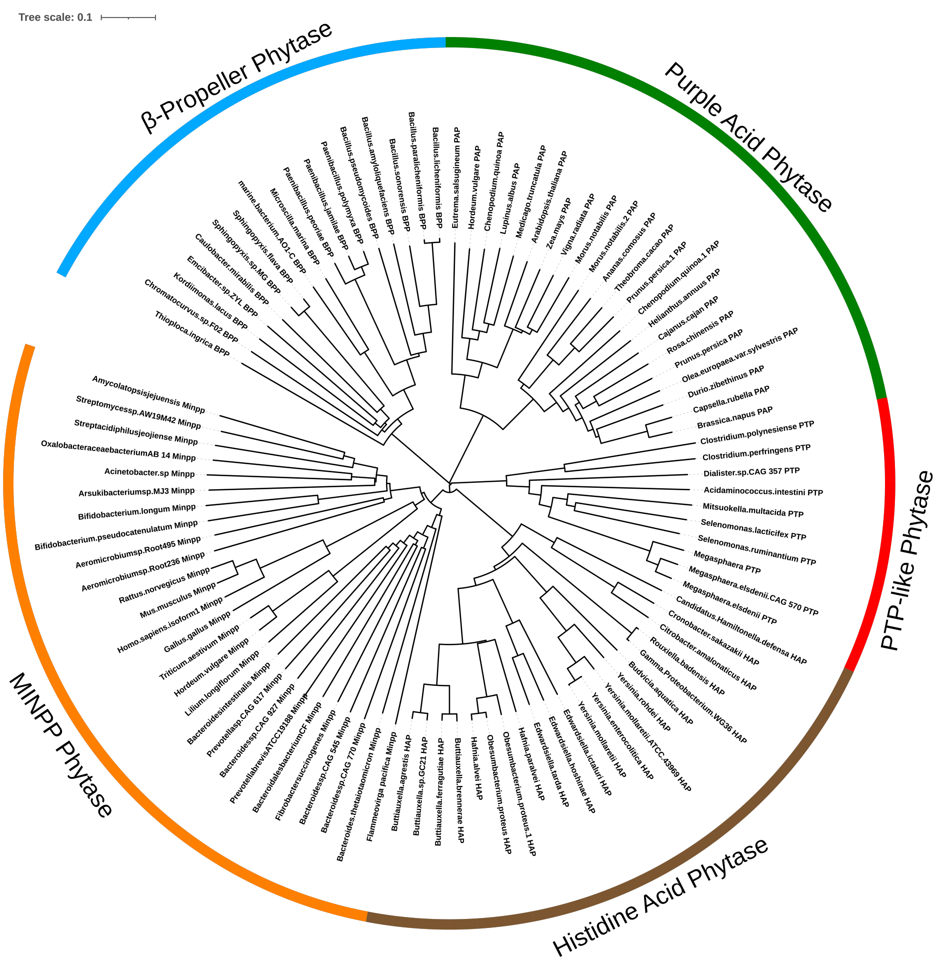

Tree scale: 0.1
β-Propeller Phytase
Purple Acid Phytase
PTP-like Phytase
Histidine Acid Phytase
MINPP Phytase
Bacillus.licheniformis BPP
Bacillus.paralicheniformis BPP
Bacillus.sonorensis BPP
Bacillus.amyloliquefaciens BPP
Bacillus.pseudomycoides BPP
Paenibacillus.polymyxa BPP
Paenibacillus.jamilae BPP
Paenibacillus.peoriae BPP
Microscilla.marina BPP
marine.bacterium.AO1-C BPP
Sphingopyxis.flava BPP
Sphingopyxis.sp.MG BPP
Caulobacter.mirabilis BPP
Emcibacter.sp.ZYL BPP
Kordiimonas.lacus BPP
Chromatocurvus.sp.F02 BPP
Thioploca.ingrica BPP
Eutrema.salsugineum PAP
Hordeum.vulgare PAP
Chenopodium.quinoa PAP
Lupinus.albus PAP
Medicago.truncatula PAP
Arabidopsis.thaliana PAP
Zea.mays PAP
Vigna.radiata PAP
Morus.notabilis PAP
Morus.notabilis.2 PAP
Ananas.comosus PAP
Theobroma.cacao PAP
Prunus.persica.1 PAP
Chenopodium.quinoa.1 PAP
Helianthus.annuus PAP
Cajanus.cajan PAP
Rosa.chinensis PAP
Prunus.persica PAP
Olea.europaea.var.sylvestris PAP
Durio.zibethinus PAP
Capsella.rubella PAP
Brassica.napus PAP
Clostridium.polynesiense PTP
Clostridium.perfringens PTP
Dialister.sp.CAG 357 PTP
Acidaminococcus.intestini PTP
Mitsuokella.multacida PTP
Selenomonas.lacticifex PTP
Selenomonas.ruminantium PTP
Megasphaera PTP
Megasphaera.elsdenii.CAG 570 PTP
Megasphaera.elsdenii PTP
Candidatus.Hamiltonella.defensa HAP
Cronobacter.sakazakii HAP
Citrobacter.amalonaticus HAP
Rouxiella.badensis HAP
Gamma.Proteobacterium.WG36 HAP
Budvicia.aquatica HAP
Yersinia.rohdei HAP
Yersinia.mollaretii.ATCC.43969 HAP
Yersinia.enterocolitica HAP
Yersinia.mollaretii HAP
Edwardsiella.ictaluri HAP
Edwardsiella.hoshinae HAP
Edwardsiella.tarda HAP
Hafnia.paralvei HAP
Obesumbacterium.proteus.1 HAP
Obesumbacterium.proteus HAP
Hafnia.alvei HAP
Buttiauxella.brennerae HAP
Buttiauxella.ferragutiae HAP
Buttiauxella.sp.GC21 HAP
Buttiauxella.agrestis HAP
Flammeovirga pacifica Minpp
Bacteroides.thetaiotaomicron Minpp
Bacteroidessp.CAG 770 Minpp
Bacteroidessp.CAG 545 Minpp
Fibrobactersuccinogenes Minpp
BacteroidalesbacteriumCF Minpp
PrevotellabrevisATCC19188 Minpp
Bacteroidessp.CAG 927 Minpp
Prevotellasp.CAG 617 Minpp
Bacteroidesintestinalis Minpp
Lilium.longiflorum Minpp
Hordeum.vulgare Minpp
Triticum.aestivum Minpp
Gallus.gallus Minpp
Homo.sapiens.isoform1 Minpp
Mus.musculus Minpp
Rattus.norvegicus Minpp
Aeromicrobiumsp.Root236 Minpp
Aeromicrobiumsp.Root495 Minpp
Bifidobacterium.pseudocatenulatum Minpp
Bifidobacterium.longum Minpp
Arsukibacteriumsp.MJ3 Minpp
Acinetobacter.sp Minpp
OxalobacteraceaebacteriumAB 14 Minpp
Streptacidiphilusjeojiense Minpp
Streptomycessp.AW19M42 Minpp
Amycolatopsisjejuensis Minpp

Figure S2.

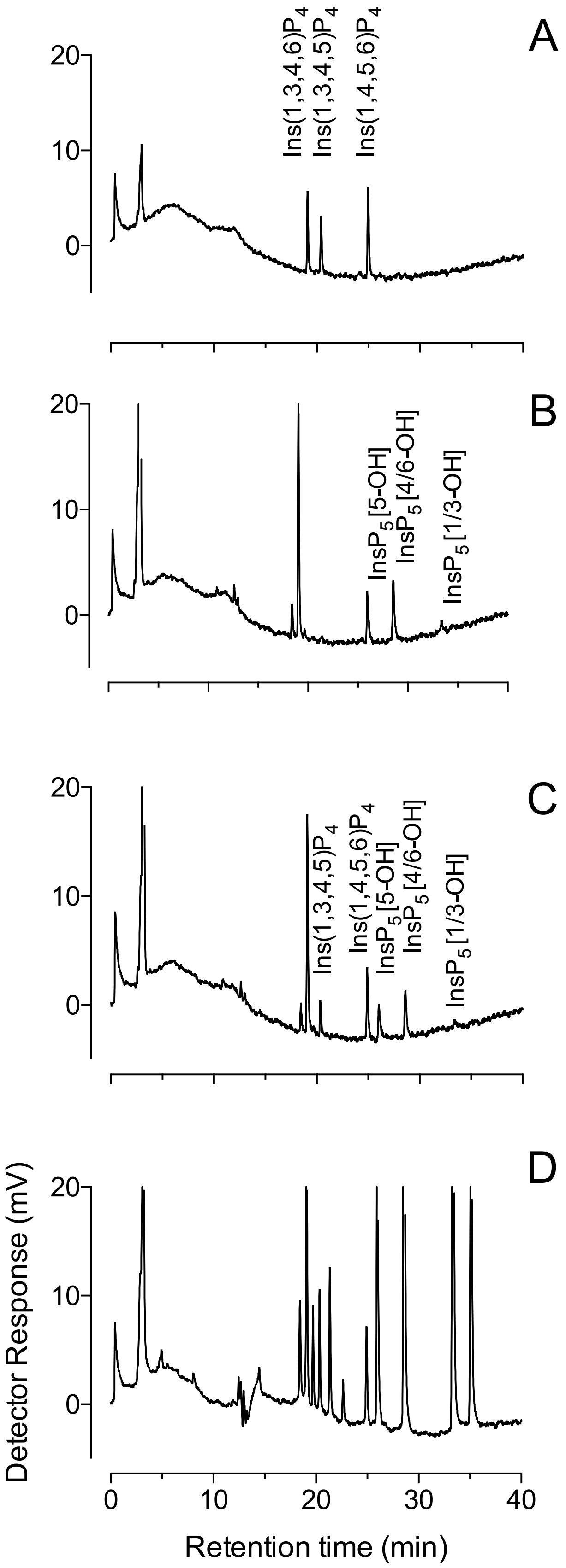
A
B
C
D
Ins(1,3,4,6)P$_4$
Ins(1,3,4,5)P$_4$
Ins(1,4,5,6)P$_4$
InsP$_5$[5-OH]
InsP$_5$[4/6-OH]
InsP$_5$[1/3-OH]
Ins(1,3,4,5)P$_4$
Ins(1,4,5,6)P$_4$
InsP$_5$[5-OH]
InsP$_5$[4/6-OH]
InsP$_5$[1/3-OH]
Detector Response (mV)
Retention time (min)
0
10
20
30
40

Figure S3.

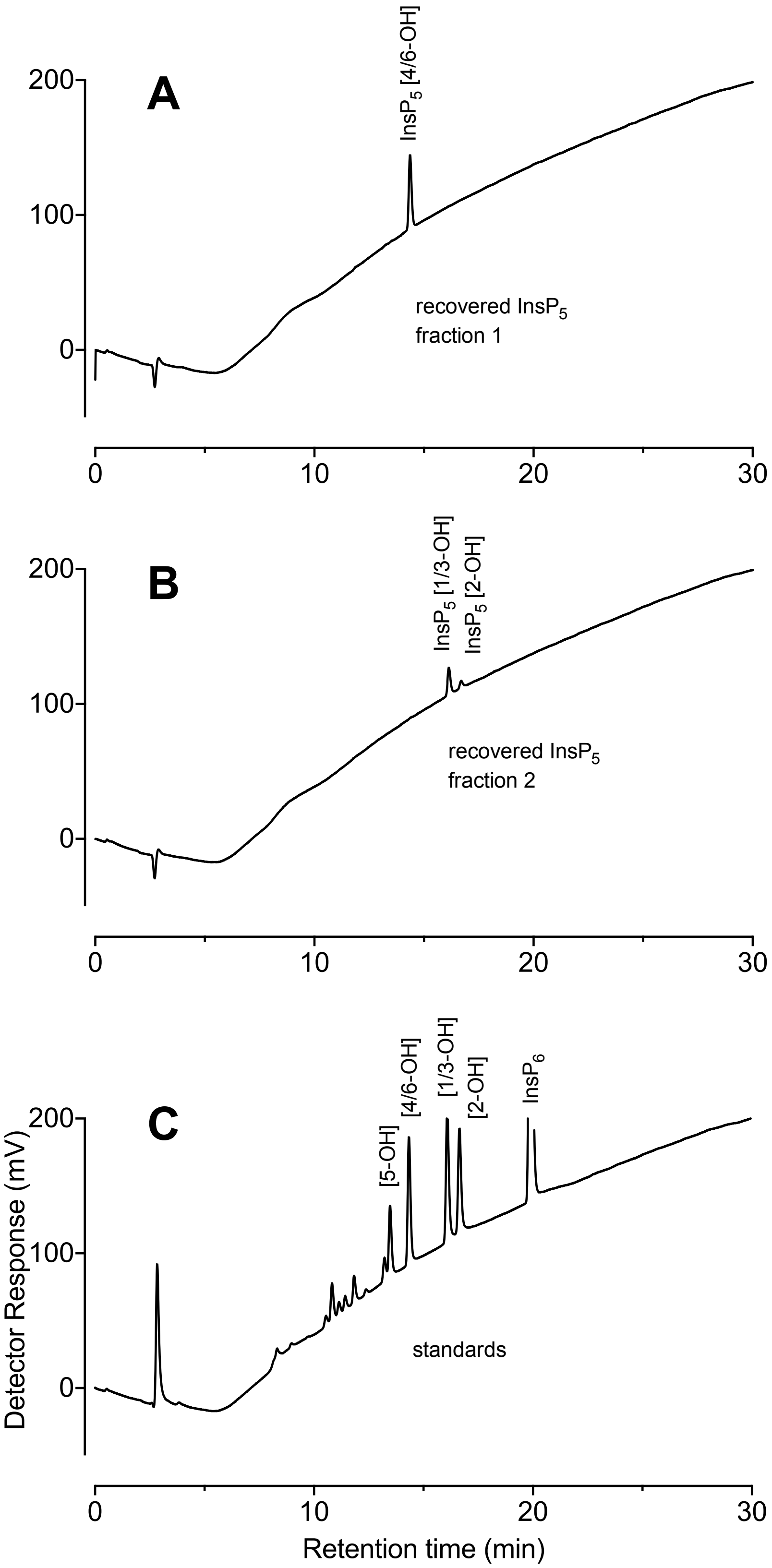

A
InsP5 [4/6-OH]
recovered InsP5
fraction 1
B
InsP5 [1/3-OH]
InsP5 [2-OH]
recovered InsP5
fraction 2
C
[5-OH]
[4/6-OH]
[1/3-OH]
[2-OH]
InsP6
standards
Detector Response (mV)
Retention time (min)
200
100
0
0
10
20
30

Figure S4.

A

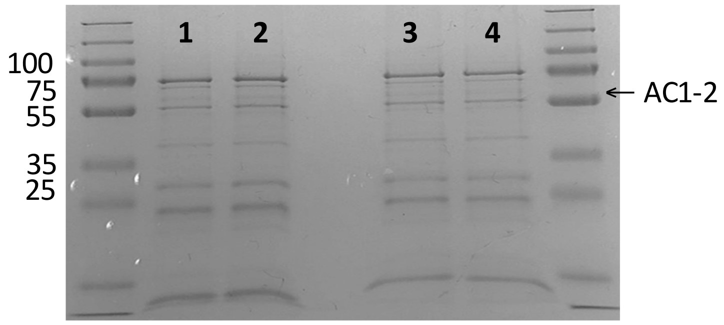
1
2
3
4
100
75
55
35
25
AC1-2

B

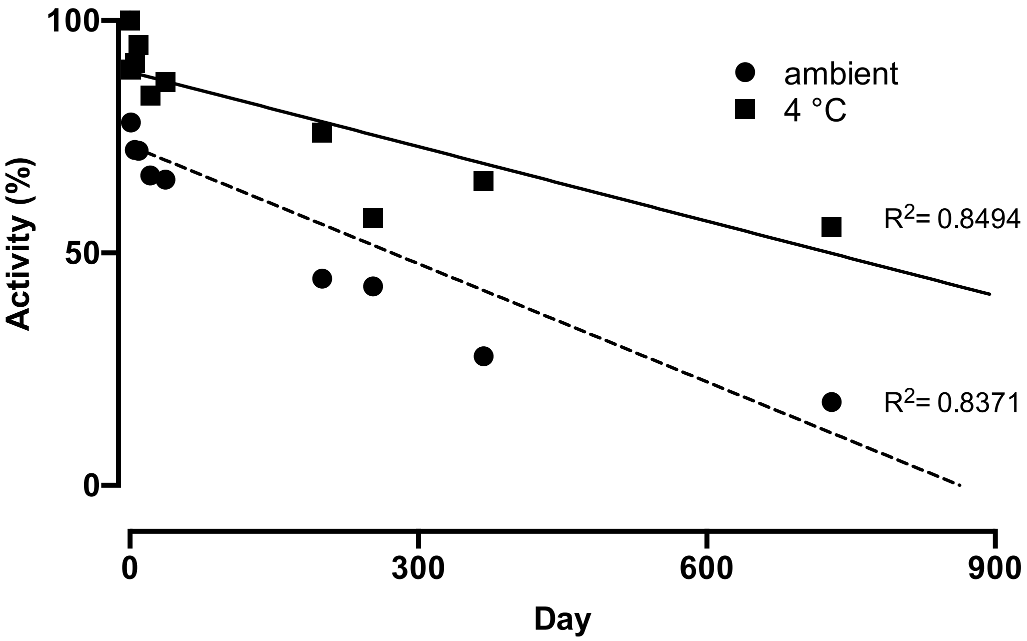
ambient
4 °C
R2= 0.8494
R2= 0.8371
Activity (%)
100
50
0
0
300
600
900
Day

Figure S5.

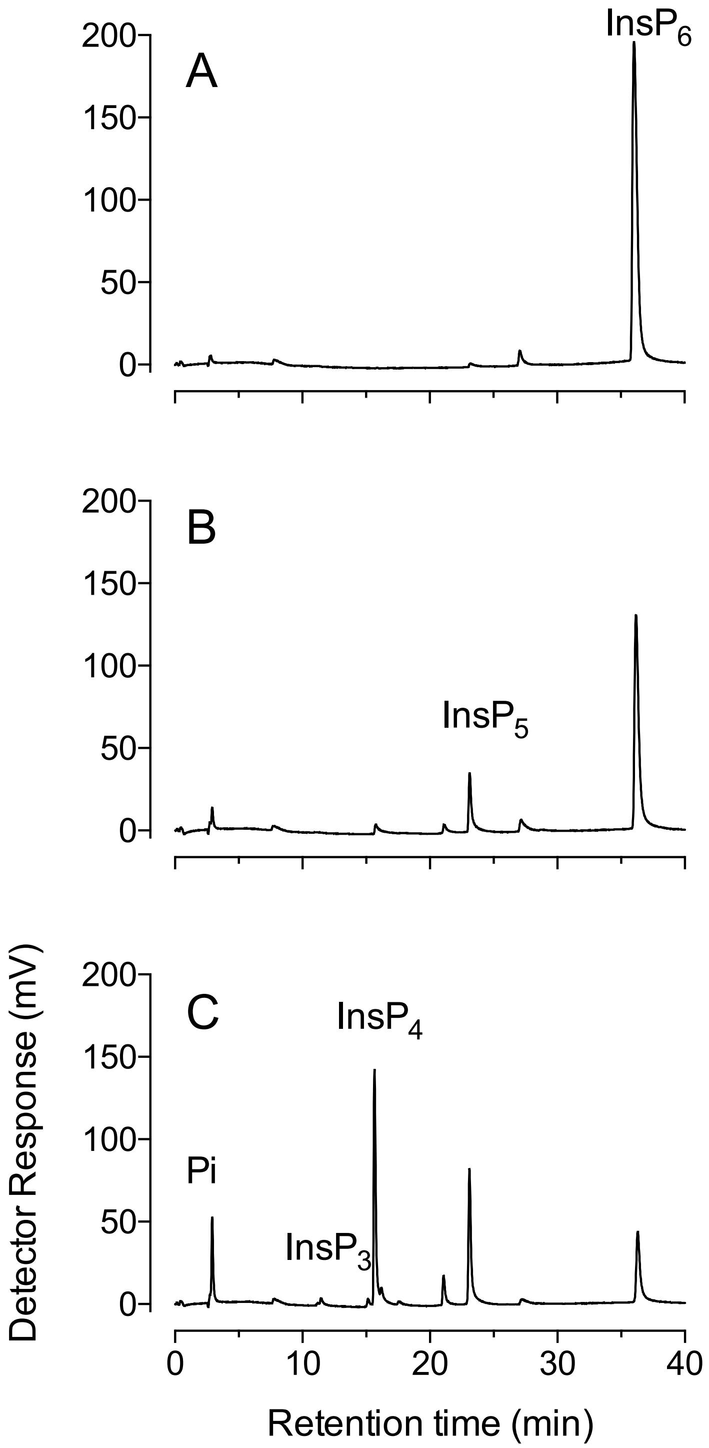

A
InsP6
B
InsP5
C
InsP4
Pi
InsP3
200
150
100
50
0
0
10
20
30
40
Detector Response (mV)
Retention time (min)
